# T cell receptor repertoire analysis in HTLV-1-associated diseases

**DOI:** 10.3389/fimmu.2022.984274

**Published:** 2022-09-15

**Authors:** Annaliese Clauze, Yoshimi Enose-Akahata, Steven Jacobson

**Affiliations:** Viral Immunology Section, National Institute of Neurological Disorders and Stroke, National Institutes of Health, Bethesda, MD, United States

**Keywords:** human T lymphotropic virus 1, adult T-cell leukemia/lymphoma, HTLV-1-associated myelopathy/tropical spastic paraparesis, T cell receptor (TCR), TCR repertoire

## Abstract

Human T lymphotropic virus 1 (HTLV-1) is a human retrovirus identified as the causative agent in adult T-cell leukemia/lymphoma (ATL) and chronic-progressive neuroinflammatory disorder HTLV-1-associated myelopathy/tropical spastic paraparesis (HAM/TSP). HTLV-1 is estimated to infect between 5-20 million people worldwide, although most infected individuals remain asymptomatic. HTLV-1 infected persons carry an estimated lifetime risk of approximately 5% of developing ATL, and between 0.25% and 1.8% of developing HAM/TSP. Most HTLV-1 infection is detected in CD4^+^ T cells *in vivo* which causes the aggressive malignancy in ATL. In HAM/TSP, the increase of HTLV-1 provirus induces immune dysregulation to alter inflammatory milieu, such as expansion of HTLV-1-specific CD8^+^ T cells, in the central nervous system of the infected subjects, which have been suggested to underlie the pathogenesis of HAM/TSP. Factors contributing to the conversion from asymptomatic carrier to disease state remain poorly understood. As such, the identification and tracking of HTLV-1-specific T cell biomarkers that may be used to monitor the progression from primary infection to immune dysfunction and disease are of great interest. T cell receptor (TCR) repertoires have been extensively investigated as a mechanism of monitoring adaptive T cell immune response to viruses and tumors. Breakthrough technologies such as single-cell RNA sequencing have increased the specificity with which T cell clones may be characterized and continue to improve our understanding of TCR signatures in viral infection, cancer, and associated treatments. In HTLV-1-associated disease, sequencing of TCR repertoires has been used to reveal repertoire patterns, diversity, and clonal expansions of HTLV-1-specific T cells capable of immune evasion and dysregulation in ATL as well as in HAM/TSP. Conserved sequence analysis has further been used to identify CDR3 motif sequences and exploit disease- or patient-specificity and commonality in HTLV-1-associated disease. In this article we review current research on TCR repertoires and HTLV-1-specific clonotypes in HTLV-1-associated diseases ATL and HAM/TSP and discuss the implications of TCR clonal expansions on HTLV-1-associated disease course and treatments.

## Introduction

Human T lymphotropic type 1 (HTLV-1) is a retrovirus known to be an etiological agent of an aggressive mature T cell malignancy termed adult T cell leukemia/lymphoma (ATL) and the chronic, progressive inflammatory neurologic disease HTLV-1-associated myelopathy/tropical spastic paraparesis (HAM/TSP) (1–4). Although most infected individuals remain asymptomatic, HTLV-1-infected persons carry an estimated lifetime risk of approximately 2-5% of developing ATL (5), and 0.25-1.8% of developing HAM/TSP (6, 7), which varies between studies and ethnic groups. Disease development such as T cell transformation and inflammation occurs after many years of chronic infection of HTLV-1. The predominant reservoir of HTLV-1 is CD4^+^ T cell in both ATL and HAM/TSP patients as well as asymptomatic carriers. Two viral proteins, Tax and HBZ, play critical roles in HTLV-1 oncogenesis and chronic inflammation. Tax is a transforming and transactivating protein of HTLV-1 and induces the expression of a variety of cellular genes by activation of the NF-κB pathway and the modulation of the epigenetic machinery to induce cellular proliferation and transformation (8). HBZ is encoded by the minus strand of the HTLV-1 provirus and ubiquitously expressed in all ATL cells and PBMCs of HTLV-1-infected subjects (9, 10). HBZ promotes proliferation and survival of ATL cells, suppresses Tax-mediated viral transcription, and inhibits the classic NF-κB pathway (8). Accumulating evidence showed that Tax and HBZ are important factors for both ATL and HAM/TSP, but it remains unknown how the virus can lead to such different diseases and why only small numbers of HTLV-1-infected individuals develop these diseases.

ATL has four clinical subtypes including acute, lymphoma, chronic, and smoldering subtypes, and its prognosis remain poor, especially in aggressive typed ATL (acute and lymphoma type) due to rapid progression (11). ATL is characterized by clonal proliferation of CD4^+^ T cells containing integrated HTLV-1 provirus, typically associated with T cell receptor (TCR) gene rearrangements (12, 13). The malignant T cells have lobulated nuclei (“flower cells”) with condensed chromatin and express characteristic T cell markers, CD3^+^, CD4^+^, CD5^+^, CD7^-^, CD25^+^, CD26^-^, and a monoclonal TCRVβ (14). Previous studies demonstrated that increase of HTLV-1 proviral load (PVL) and clonal expansion of HTLV-1-infected cells in PBMCs reflect a high risk of ATL transformation in HTLV-1-infected subjects (15, 16). Interestingly, while about 60% of ATL patients lost the *tax* gene expression, the *HBZ* gene is expressed in all ATL cells (10, 17). HBZ has been demonstrated to induce similar immunophenotypes of ATL cells in HBZ-transgenic mice, suggesting that HBZ plays an important role for proliferation and infiltration of ATL cells (18, 19). Both Tax and HBZ are immunogenic proteins recognized by HTLV-1-specific cytotoxic CD8^+^ T cells (CTL) which plays a crucial role in immunity against HTLV-1 to secrete various factors that suppress viral replication and kill infected target cells (20–24). However, HBZ-specific CTL were less detectable in HTLV-1-infected subjects compared to Tax-specific CTL (20, 23, 24). Moreover, the frequency and function of HTLV-1-specific CTL are reduced in ATL patients (25, 26). Therefore, the immunologic effect against ATL cells may play a critical role in the prevention of ATL development (27).

HAM/TSP is a progressive, chronic inflammatory myelopathy of the central nervous systems (CNS) (28). HTLV-1 PVL in PBMCs is higher in HAM/TSP than in asymptomatic carriers (29) and has been shown to be significantly elevated in HAM/TSP cerebrospinal fluid (CSF) cells than in PBMCs (30, 31). These observations have led to the hypothesis that an increased HTLV-1 PVL is associated with an increased risk of HAM/TSP disease progression. It has been demonstrated that HTLV-1-infected CD4^+^ T cells can induce the production of proinflammatory cytokines and proliferation of CD8^+^ T cells (32–34). CD8^+^ T cells, including HTLV-1 Tax11-19-specific CTL which recognize an immunodominant HTLV-1 Tax antigen particularly in patients who are HLA-A*0201, are found in high numbers in PBMCs, even higher in the CSF. This corelates with the increased levels of HTLV-1-infected lymphocytes and high PVL in both compartments (21, 35–39). This higher frequency of CD8^+^ T cells and HTLV-1 PVL in CSF has recently been shown to have clinical consequence since a quantitative radiological analysis of the spinal cord has shown a correlation of spinal cord atrophy in HAM/TSP associated with increased CD8^+^ T cells (40). It remains a question why HAM/TSP patients have high PVL despite vigorous HTLV-1-specific CTL, while a strong, chronically activated CTL response to HTLV-1 was found in both asymptomatic carriers and HAM/TSP patients (41–43). Several reports have demonstrated the mechanism in HAM/TSP patients including dysregulation in CD4^+^ regulatory T cells (44), degenerate specificity and exhaustion in HTLV-1-specific CD8^+^ T cells (45–50), and increased T cell proliferation due to high expression of the common γ chain family of cytokines and their receptors, such as IL-2 and IL-15, associated with HTLV-1 gene transactivation (51, 52). The presence of both HTLV-1-infected lymphocytes and chronically activated CD8^+^ T cells in high numbers in the CSF generating a highly proinflammatory environment support the hypothesis that HAM/TSP is an immunopathologically mediated disease associated with bystander damage to surrounding oligodendrocytes (53).

In both HAM/TSP and ATL, sequencing of TCR repertoires has been a useful tool in parsing the drivers of disease. As such, identification of biomarkers for an HTLV-1-associated disease outcome is a central focus in the field. Advances in high throughput and single cell RNA sequencing technology have made characterization of TCR repertoires more efficient, economical, and accessible in recent years, and offer unmatched resolution of T cell populations. Unique molecular identifier (UMI) based single cell sequencing techniques have been used to identify unique molecular reads and remove bias introduced by PCR amplification. This technique has allowed highly accurate quantitation of T cell clonal expansions in disease states. Additionally, sequencing of the complementary determining region 3 (CDR3), responsible for peptide recognition has been used to determine HTLV-1 and disease-specific motifs in both HAM/TSP and ATL. In this review, we will compare the differing TCR repertoire profiles of HAM/TSP and ATL and discuss differences in profiles that may contribute to differing disease outcomes.

## TCR repertoire profiles in healthy individuals

**Figure 1:**
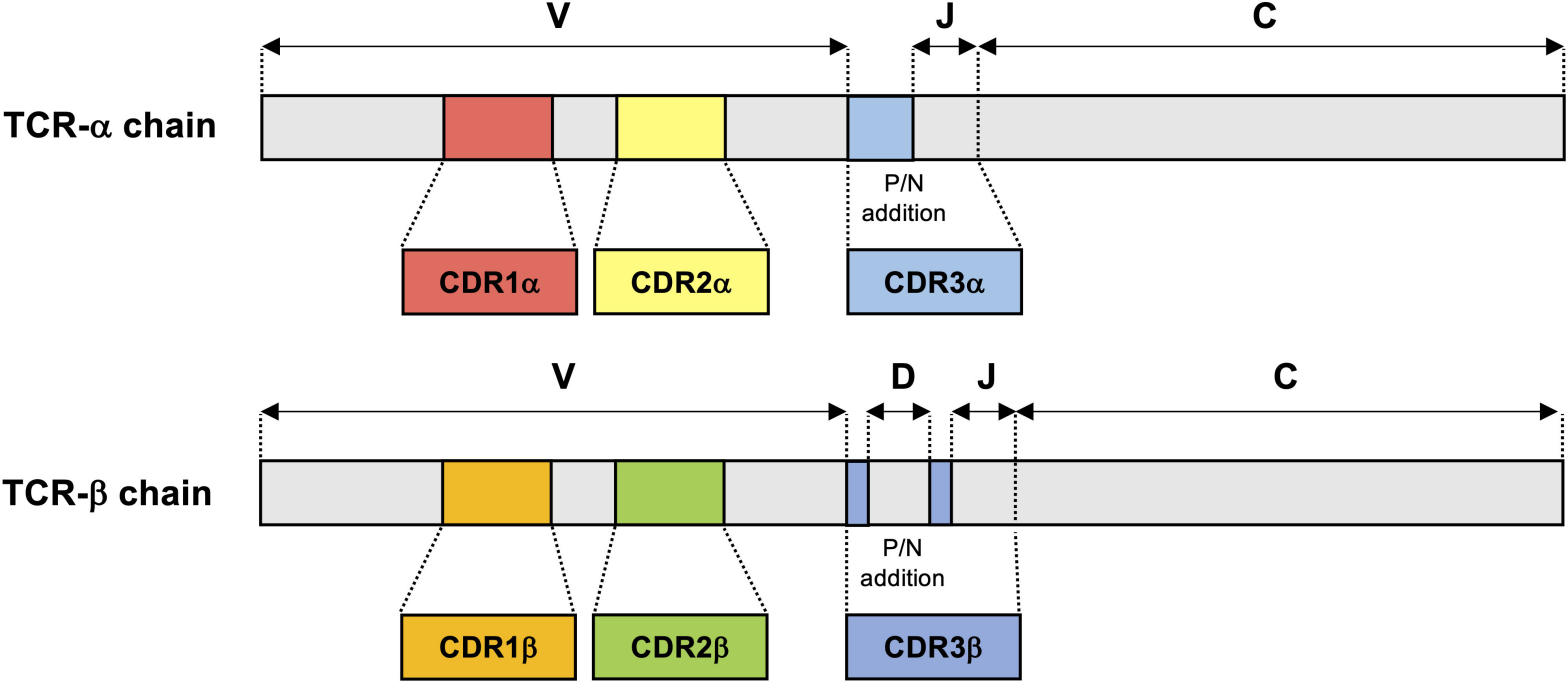
TCR-α and TCR-β chain gene structure. TCR-α and TCR-β chains consist of a variable (V) amino-terminal region and a constant (C) region. The complementarity-determining region 1 (CDR1) and CDR2 encoded in the TCR germline V genes, which are conserved across TCR-α and TCR-β. In contrast with CDR1 and CDR2, CDR3 is located at the junction between the rearranged V and joining (J) segments in TCR-α chain and V, diversity (D) and J segments in TCR-β chain. Additions of P- and N-nucleotides (P/N addition) are present in the junctions between the V, D, and J gene segments of the rearranged TCR-β chain and also between the V and J gene segments of all rearranged TCR-α chain.

Diversity of TCR repertoires is an essential characteristic of the highly adaptable healthy immune system; a highly polyclonal repertoire allows the immune system to quickly mount responses to a wide variety of antigens (54). TCR diversity is determined by the combination of the variable (V), diversity (D), and joining (J) regions of TCR genes, as well as the introduction of random insertions and deletions at gene junctions (**Figure 1**) (55, 56). TCR repertoire analysis has mainly relied on TCR β chain sequences due to several reasons, such as the presence of the D gene component in TCR-β and unique expression of TCR-β on each single αβ T cell, but recent advance of single cell TCR sequencing approaches can identify the pairs of α and β chains to provide more accurate TCR diversity and the biological function (57). Estimates of total possible unique TCR sequences range from 10^15^ to 10^20^ in PBMCs (56, 58), with naive T cell subsets exhibiting a very low frequency of individual clones and memory T cell subsets exhibiting more clonal expansions in TCR-α and TCR-β chains (59). In healthy individuals, peripheral blood TCR repertoires have been observed to be highly polyclonal, demonstrate some stability over time, and are highly specific to the individual in which TCR-β sequences in particular are highly conserved, although identical sequences may rarely be found between patients (60, 61). Within the healthy individual’s repertoire, small subpopulations of 1-5% of the total TCR-β sequences are observed to be persistent over time, perhaps indicating immunological selection pressures in response to low-grade chronic antigen exposures. These persistent receptor sequences are encoded by an increased number of redundant nucleotide sequences and make up a significantly larger proportion of “public” TCR sequences shared across individuals than unique receptor sequences (61). By comparison, studies of the TCR repertoire in compartmentalized locations such as CSF are limited. Studies have shown very low TCR-β richness and diversity in the CSF of healthy individuals and subjects without any neuroinflammatory disease (62, 63), suggesting a lack of antigenic stimuli to drive migration and expansion of T cells within healthy CSF.

## TCR repertoire analysis in HAM/TSP

### HTLV-1-specific CD8^+^ T cells in HAM/TSP

**Figure 2:**
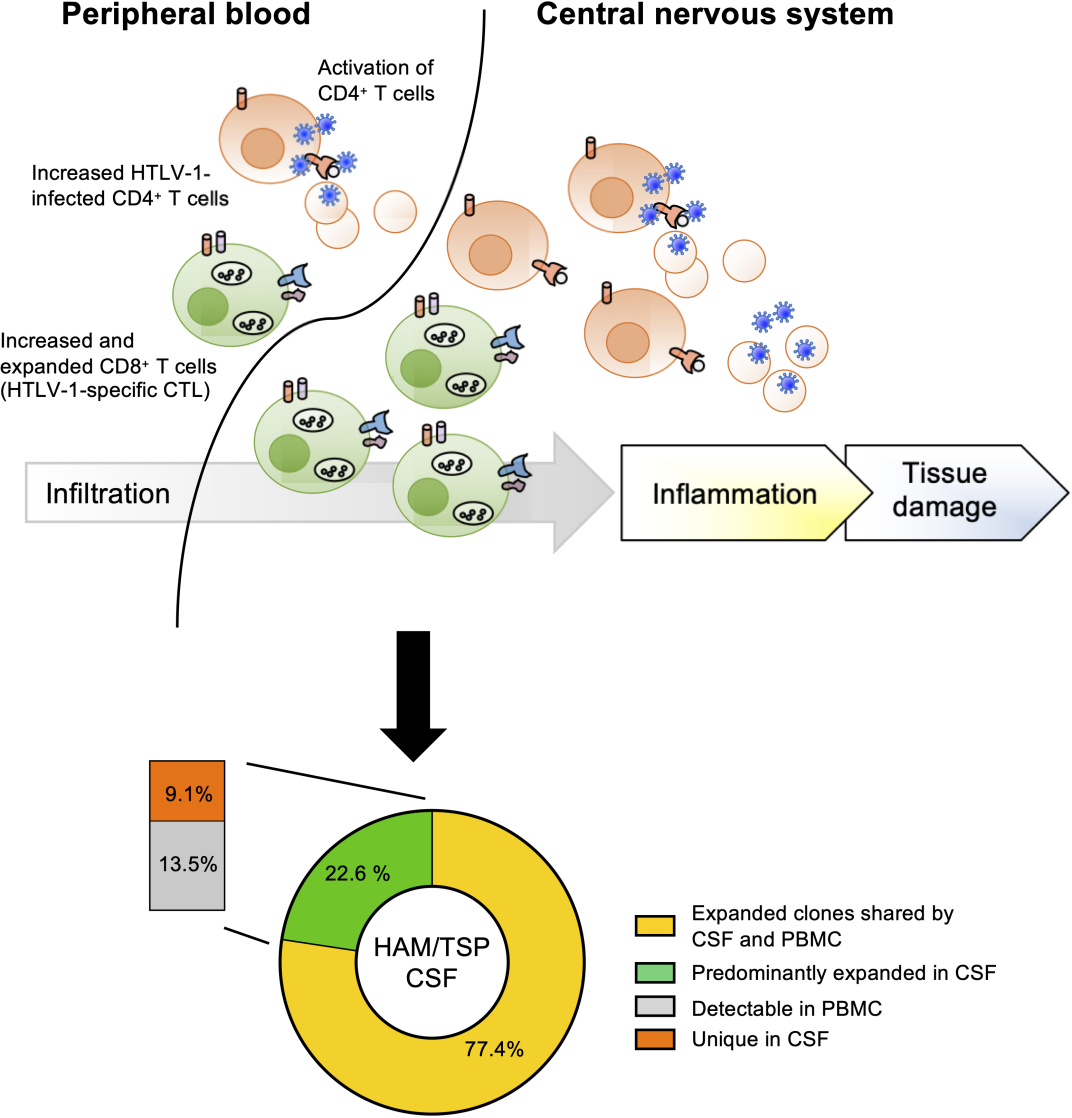
Origin of TCR expanded clones in CSF of HAM/TSP patients. HAM/TSP patients had a higher clonal T cell expansion, especially CD8^+^ T cells (including HTLV-1-specific CTL), in PBMCs as well as in CSF. Within expanded T cell clones in CSF of HAM/TSP patients, most expanded CSF TCR-β clonotypes were derived from expanded T cell clones in PBMCs (yellow). A small but distinct fraction of these expanded TCR-β clonotypes were intrathecally enriched in CSF of HAM/TSP patients [reference (37)].

In HAM/TSP, a previous report on the TCR analysis demonstrated that clonal expansion of both CD4^+^ and CD8^+^ T lymphocytes occurs in both asymptomatic carriers and HAM/TSP patients and that the total number of expanded clones in the CD8^+^ T lymphocyte population was much greater than that of the CD4^+^ T lymphocytes (64). Using technology based on high-throughput sequencing and bioinformatics methods, it has been recently shown that HAM/TSP patients had a higher clonal T cell expansion in PBMCs as well as purified CD4^+^ and CD8^+^ cells compared with healthy individuals and patients with multiple sclerosis, a clinically similar disease to HAM/TSP but whose etiologic trigger has not yet been identified (60). In addition, both cross-sectional and longitudinal analysis of TCR-β clonal expansions in HAM/TSP patients have shown significant correlation with HTLV-1 PVL and increased effector/memory and effector phenotypes in both CD4^+^ and CD8^+^ T cell subsets in PBMCs (60, 63), suggesting that clonal expansions closely reflect active immune response to HTLV-1 infection. Moreover, sequencing analyses of TCR-β repertoires demonstrated significantly greater oligoclonal expansion in CSF compartments as well as PBMCs of HAM/TSP patients compared to healthy individuals (63). The clonal expansion of TCR-β clonotypes in CSF of HAM/TSP patients has been suggested to be associated with high numbers of activated CD4^+^ T cells and CD8^+^ T cells found in CSF of HAM/TSP patients (34, 35, 65, 66). Importantly, while a large fraction (77.4%) of expanded TCR-β clones identified in the CSF of HAM/TSP patients were also demonstrated in matched PBMC, the other 22.6% of expanded clones in the CSF that were not identified in matched PBMCs appeared to be specific for the CSF compartment (**Figure 2**) (63). Collectively these results indicate an antigen-driven immune response in which the majority of TCR clones in the CSF are derived from the periphery, while a small but distinct population of clones are the result of intrathecal enrichment (63).

Compared to asymptomatic carriers, HAM/TSP patients showed significantly high levels of circulating HTLV-1-specific CD8^+^ T cells, which was able to infiltrate in CSF and spinal cord lesion of HAM/TSP patients (21, 53, 67). Selective enrichment of HTLV-1-specific CD8^+^ T cells in CSF of HAM/TSP patients strongly suggests that these cells maybe directly involved in the pathogenesis of HAM/TSP. Characterization of TCR repertoire and usage in HLA-A*0201^+^ HAM/TSP patients demonstrated that TCRs used within each patient display a limited heterogeneity, indicating an oligoclonal expansion of HTLV-1-specific CD8^+^ T cells (68, 69). Using a high-throughput sequencing technology, it has recently been demonstrated that expanded TCR clones in PBMCs of HAM/TSP patients are found in even greater proportions in CD8^+^ T cells, and more specifically, HTLV-1 Tax11-19-specific CD8^+^ T cells (63). However, as the highest ranking TCR-β clonotypes in the peripheral blood did not appear to be used by HTLV-1 Tax11-19-specific CD8^+^ T cell clones (63), it suggests that the peripheral blood compartment may not best reflect the antigen (HTLV-1) driven immunological responses characteristic of the disease. Rather, analysis of TCR-β clonotypes from Tax11-19-specific CD8^+^ T cells in the CSF of one HAM/TSP patient with matched PBMC data showed seven TCR-β sequences shared between PBMCs and CSF (63). Of these seven TCR-β sequences, two clones from Tax11-19-specific CD8^+^ T cells were detected more in the CSF than PBMCs, suggesting that a subset of Tax11-19-specific TCR-β clonotypes was clonally expanded in peripheral blood and was subsequently infiltrated and becoming highly enriched in the CSF (63). These observations are consistent with the hypothesis that HAM/TSP is immunopathologically mediated by HTLV-1-specific CTL whose TCR-β clonotypes can be demonstrated to be expanded in the CSF.

### CD4^+^ T cells in HAM/TSP

In contract to CD8^+^ T cells, little is known about TCR repertoire in CD4^+^ T cells of HAM/TSP patients, although TCR clonal expansion was detected in both CD4^+^ and CD8^+^ T cells in HAM/TSP patients (60, 64). Using flow cytometry, comparison of TCR Vβ usage showed that TCR Vβ7.2 was under-utilized and Vβ12 was over-utilized in CD4^+^ T cells of HTLV-1-infected individuals compared with healthy uninfected controls, whereas there were no such differences in CD8^+^ T cells (70). While the virological and immunological events are different between HAM/TSP and ATL, the frequency of ATL development in HAM/TSP patients is extremely rare and has been reported to be approximately 3.81 per 1000 person-years (71). Since HTLV-1-infected subjects including HAM/TSP patients may be at risk for developing ATL, further studies about TCR repertoire analysis of CD4^+^ T cells would be needed for a more complete understanding of CD4^+^ T cell dynamics in HTLV-1-infected subject.

## TCR repertoire analysis in ATL

### Malignant T cells

Malignant T cells in ATL patients are derived from clonally expanded T cells with HTLV-1 provirus integrated into the cellular genome and express characteristic T cell markers, CD3^+^, CD4^+^, CD5^+^, CD7^-^, CD25^+^, CD26^-^, CCR4^+^, CADM-1^+^ and monoclonal TCR Vβ (14, 72–76). Using high throughput sequencing, TCR repertoire analysis in PBMCs demonstrated that ATL patients showed oligo- or monoclonal patterns of TCR clonotypes whereas asymptomatic carriers and healthy individuals showed polyclonal patterns (77, 78). However, expression of TCR-α and TCR-β genes in the dominant clone differed among the samples (77). The most and commonly expressed TCR-β clone constituting 60-99% of total clones, while the clonal percent of the top TCR-β clone from non-malignant HTLV-1-associated disease patients ranges from 1% to 40%, highlighting the stark differences in ATL clonal profiles (79). In ATL, four clinical subtypes (acute, lymphoma, chronic and smoldering) have been identified, which range from highly aggressive to indolent in their clinical course (11). Some studies have demonstrated significant variation in ATL TCR repertoire based on disease subtype in which smoldering ATL patients showed significantly higher TCR diversity compared with the other subtypes while diversity significantly decreased in more aggressive stages of the disease, including acute, chronic, and lymphoma types (77, 78).

Importantly within ATL, TCR-β clonal expansions originate primarily from HTLV-1-infected cells. Characterization of TCR repertoire together with virological approaches, such as genomic and viral transcriptomes, provirus integration, and somatic mutation, are essential to understanding the heterogeneity and complexity of ATL. Using high throughput genome sequencing and flow cytometric screening of TCR repertoire and T cell markers, longitudinal analysis of TCR clones in one smoldering ATL patient revealed a gradual switch from one dominant HTLV-1-infected T cell clone (Vβ 20) to another (Vβ 13.1), corresponding to an increase in somatic mutations associated with upregulation of genes downstream of the TCR pathway (80). Further longitudinal studies of the TCR repertoire in ATL may offer insight into the relationship between progression of ATL disease subtype and shifts in dominant clones. In some cases of ATL, dominant TCR clones were skewed, with only dominant CDR3 TCR-α or TCR-β sequences being observed in a single individual (77). As both TCR-α and TCR-β sequences are typically expressed in conjunction and necessarily must interact with CD3 for the differentiation and survival of T cells, these skewed TCR clones may coincide with previous findings of reduced CD3 protein expression in ATL (81). A recent advance of TCR repertoire analysis with multiple bioinformatics analysis at single cell level also demonstrated that HTLV-1-infected cells in an activated state further transformed into ATL cells, which are characterized as clonally expanded, highly activated T cells (78). In addition, while healthy individuals harbored T cells with an activated phenotype, in ATL, infected T cells and ATL cells became spontaneously activated, acquired a regulatory T cell phenotype, and subsequently progressed to a state of extreme activation, which was maintained throughout the ATL phase (78). Thus, new technologies and bioinformatics tools at single cell level will further improve our ability to identify mechanistic pathways responsible for the *in vivo* transformation of HTLV-1-infected T cells into leukemic cells.

### Malignant T cell HTLV-1-specific CD8^+^ T cells in ATL

In contrast with HAM/TSP, ATL patients are commonly immunosuppressed and have a lower frequency and diversity of HTLV-1-specific CD8^+^ T cells (11, 25, 26, 82, 83). TCR repertoire analysis in CD8^+^ T cells have been studied for anti-viral T cell-based treatment of ATL. Previous studies demonstrated that HTLV-1 Tax-specific CD8^+^ T cells could prevent relapse in ATL patients who have undergone allogeneic hematopoietic stem cell transplantation (allo-HSCT) (84, 85). In addition, Tax301-309-specific CD8^+^ T cells were increased in ATL patients who achieved complete remission after allo-HSCT and TCR repertoires in Tax301-309-specific CD8^+^ T cells of ATL patients were highly restricted having a particular amino acid sequence motif (PDR) in CDR3 of the TCR-β chain (86–88).

### TCR repertoire and treatment of ATL

In addition to allo-HSCT which can achieve long-term remission, treatments of ATL may additionally affect TCR clonal profiles and act as a marker of remission. Following treatment of ATL with traditional chemotherapy (mLSG15) which can provide short-term survival of about one year (14), major TCR clonotypes were reduced but remained dominant within the repertoire, and typical polyclonal T cells were not fully reestablished (89). Mogamulizumab is a humanized anti-CCR4 antibody to kill CCR4^+^ cells by enhanced antibody-dependent cellular cytotoxicity and has shown substantial anti-ATL activity, even in relapsing or chemotherapy-resistant disease (90, 91). Mogamulizumab treatment of ATL resulted in the reduction of ATL-associated TCR clones and a return to polyclonal repertoire in CD4^+^ T cells and oligoclonal repertoire in CD8^+^ T cells, suggesting remarkable reduction or elimination of clonal cells, and enhanced reconstitution of non-tumor polyclonal CD4^+^ T cells and oligoclonal CD8^+^ T cells (89). Thus, TCR repertoire analysis can provide strong insights to understand immune reconstitution in ATL patients undergoing anti-tumor treatment.

## Conserved TCR motifs

In HTLV-1-associated disease, identification of conserved TCR sequences or motifs maybe useful in predicting disease-specific outcomes in early-stage HTLV-1 infection. While it is still challenging to identify shared TCR sequences between individuals and disease-specific TCRs across patients (92), sequence motif analysis may offer insights into key antigen determinants that maybe predictive of an HTLV-1-associated disease outcome.

### HAM/TSP

The amino acid sequencing analysis in the CDR3 region of TCR demonstrated the shared amino acid motif in the CDR3β in CD8^+^ T cells and Tax11-19-specific CD8^+^ T cells in HAM/TSP patients (64, 93, 94). Recently, amino acid CDR3 repertoire analysis of expanded clones in Tax11-19-specific CD8^+^ T cells from HAM/TSP patients with HLA-A*0201 has demonstrated a consensus sequence of interest. While exact TCR-β sequences amongst HLA-A*0201 patients are largely private, an amino acid sequence motif, PGLAG, at position 4-8 of the CDR3 region, was found in over half of sequenced HAM/TSP patients (63). These findings are consistent with previous indications of a possible PG or PXG CDR3 motif, which were found in 50% of HLA-A*0201 HAM/TSP patients (64). In addition, it has been demonstrated that similar motifs, such as PGL at positions 5-7 and SLG at position 8-10, in the center of the CDR3 region were detected in some expanded TCR-β clonotypes in CSF of HAM/TSP patients (63). Since HAM/TSP patients have the complexity of T cell expansions including both CD4^+^ and CD8^+^ T cells (60, 63, 64, 93, 94), it would be important to identify TCR motifs that reflect disease and/or pathogen specificity and local inflammation.

### ATL

A particular amino acid motif, the PDR sequence found at position 108-110 in CDR3β of Tax301-309-specific TCRs, has been found to be highly conserved between asymptomatic carriers and ATL patients with HLA-A*24:02 (86–88). In addition, Tax301-309-specific CD8^+^ T cells of asymptomatic carriers and ATL patients commonly showed highly restricted TCR repertoires with a strongly biased usage of the BV7 gene family, and the preference for BV7 of Tax301-309-specific CD8^+^ T cells tended to decrease in asymptomatic carriers to ATL (88). Expression of the PDR^+^ Tax301-309-specific clones was detected in both asymptomatic carriers and ATL patients (chronic and acute subtypes) demonstrating that PDR amino acid sequence motif was conserved in CDR3β of Tax301-309-specific CD8^+^ T cells regardless of clinical subtype in HTLV-1 infection (88). Interestingly, following allo-HSCT, Tax301-309-specific PDR^+^ TCRs persisted in ATL patients (87). Longitudinal analysis of TCR repertoire in one such post-allo-HSCT ATL patient demonstrated persistence and selective expansion of PDR^+^ Tax301-309-specific TCR clones up to 3 years post-transplant and were found to exhibit strong Tax301-309-specific cytotoxic activity in peripheral blood and bone marrow (86). CTL activity of Tax301-309-specifc PDR^+^ TCR clones was importantly found to be restricted to HTLV-1-infected cells and had no effect on uninfected normal cells, regardless of autologous or allogeneic origin (95). Recently, it has been reported that HLA-A*24:02^+^ healthy individual T cells transduced with PDR^+^ TCRs have strong Tax301-309-specific reactivity against HTLV-1-infected cell lines and some ATL primary cells (96).

In addition, treatment with Tax301-309-specific PDR^+^ TCRs in NOD/Shi-scid, IL-2Rγ^null^ (NOG) mice inoculated with an HLA-A*24:02^+^ HTLV-1-infected cell line (MT-2) resulted in significant decreases in size and eventual eradication of tumors, compared to uncontrolled tumor growth and eventual death of non-genetically modified PBMC treated and control mice (96). These results collectively indicate a potentially promising therapeutic directed at TCR-β clones containing the PDR motif against HTLV-1-infected and ATL cells. In addition, since the downregulation of Tax expression or acquisition of viral and cellular gene mutations also linked to immune evasion during ATL disease course (97, 98), further analysis of therapeutic targets for ATL cells such as HBZ, is warranted.

## Conclusions

Analysis of TCR repertoires associated with a wide range of cancers, autoimmune, and inflammatory diseases in various compartments such as peripheral blood and CSF will greatly contribute to our understanding of the role that T cells play in these disorders and may lead to identification of markers of disease and even potential therapeutic targets. While understanding of TCR repertoires in HTLV-1-associated disease has made great strides in recent years due to advances in sequencing technology, large gaps remain in the literature regarding the progression of the TCR repertoire from HTLV-1-infected asymptomatic carrier to diseased individuals. Increasing evidence indicates T cell clonal profiles and motif sequences may be unique to different HTLV-1-associated diseases. Timing of progression from asymptomatic carrier to disease may improve our ability for early diagnosis and intervention. Longitudinal studies following asymptomatic carriers through disease development, treatment and remission or disease progression will be invaluable in this effort. Further studies utilizing new techniques in high throughput single cell sequencing are warranted to better characterize the *in vivo* TCR repertoire in ATL and HAM/TSP, particularly in compartments such as CSF for HAM/TSP in which large numbers of cells are difficult to obtain and which may be more closely reflective of events in the CNS rather than T cells in the peripheral blood. Lastly, TCR dynamics, tracking and mRNA single cell sequencing in local tissues would offer a valuable tool to discover antigen specificity, transcriptional profiling, and the molecular mechanisms of T cell plasticity to understand the heterogeneity and complexity of HTLV-1-associated diseases.

## Author contributions

AC and YE-A contributed to paper writing. SJ supervised and contributed to discussion and writing. All authors contributed to the article and approved the submitted version.

## Funding

This research was supported by the Intramural Research Program of the NIH, NINDS.

## Conflict of interest

The authors declare that the research was conducted in the absence of any commercial or financial relationships that could be construed as a potential conflict of interest.

## Publisher’s note

All claims expressed in this article are solely those of the authors and do not necessarily represent those of their affiliated organizations, or those of the publisher, the editors and the reviewers. Any product that may be evaluated in this article, or claim that may be made by its manufacturer, is not guaranteed or endorsed by the publisher.
